# A robust (re-)annotation approach to generate unbiased mapping references for RNA-seq-based analyses of differential expression across closely related species

**DOI:** 10.1186/s12864-016-2646-x

**Published:** 2016-05-24

**Authors:** Montserrat Torres-Oliva, Isabel Almudi, Alistair P. McGregor, Nico Posnien

**Affiliations:** Georg-August-Universität Göttingen, Johann-Friedrich-Blumenbach-Institut für Zoologie und Anthropologie, Abteilung für Entwicklungsbiologie, GZMB Ernst-Caspari-Haus, Justus-von-Liebig-Weg 11, 37077 Göttingen, Germany; Göttingen Center for Molecular Biosciences (GZMB), GZMB Ernst-Caspari-Haus, Justus-von-Liebig-Weg 11, 37077 Göttingen, Germany; Department of Biological and Medical Sciences, Oxford Brookes University, Gipsy Lane, Oxford, OX3 0BP UK; Andalusian Centre of Developmental Biology, carretera de Utrera, km.1, 41013 Seville, Spain

**Keywords:** RNA-seq, Annotation, Differential gene expression, EXONERATE, *Drosophila*, Closely related species, Emerging model systems, RPKM, DESeq2, Voom, Limma, Length bias

## Abstract

**Background:**

RNA-seq based on short reads generated by next generation sequencing technologies has become the main approach to study differential gene expression. Until now, the main applications of this technique have been to study the variation of gene expression in a whole organism, tissue or cell type under different conditions or at different developmental stages. However, RNA-seq also has a great potential to be used in evolutionary studies to investigate gene expression divergence in closely related species.

**Results:**

We show that the published genomes and annotations of the three closely related *Drosophila* species *D. melanogaster, D. simulans* and *D. mauritiana* have limitations for inter-specific gene expression studies. This is due to missing gene models in at least one of the genome annotations, unclear orthology assignments and significant gene length differences in the different species. A comprehensive evaluation of four statistical frameworks (DESeq2, DESeq2 with length correction, RPKM-limma and RPKM-voom-limma) shows that none of these methods sufficiently accounts for inter-specific gene length differences, which inevitably results in false positive candidate genes. We propose that published reference genomes should be re-annotated before using them as references for RNA-seq experiments to include as many genes as possible and to account for a potential length bias. We present a straight-forward reciprocal re-annotation pipeline that allows to reliably compare the expression for nearly all genes annotated in *D. melanogaster*.

**Conclusions:**

We conclude that our reciprocal re-annotation of previously published genomes facilitates the analysis of significantly more genes in an inter-specific differential gene expression study. We propose that the established pipeline can easily be applied to re-annotate other genomes of closely related animals and plants to improve comparative expression analyses.

**Electronic supplementary material:**

The online version of this article (doi:10.1186/s12864-016-2646-x) contains supplementary material, which is available to authorized users.

## Background

Comparative studies of gene expression have been used to understand the regulation of a wide range of biological processes. With the development of next generation sequencing (NGS) technologies, and in particular the use of Illumina sequencing platforms, reliable genome wide comparison of gene expression between different biological conditions has become possible [1–3]. Moreover, a growing number of available genome and transcriptome sequences [4–8] now provides the opportunity to compare gene expression not only in well-established, but also in emerging model systems. Especially, the comparison of gene expression between both closely [9–16] and distantly related species [17–20] has great potential to help understand phenotypic divergence and species adaptations at a mechanistic level [21].

Experiments to study differential gene expression using NGS technologies (RNA-seq) are based on a sequencing library generated from reverse transcribed messenger RNA (mRNA) that is extracted from the tissue and conditions of interest. Illumina sequencing, for example, results in the generation of millions of short reads ranging from 36 bp to 150 bp [22, 23]. The first step of the bioinformatics analysis is to align these reads to a reference that represents all transcripts that should be quantified [24–28]. This reference can be a whole genome sequence with annotated gene models or a transcriptome. The latter can either be generated by a *de novo* assembly of the RNA-seq reads [29, 30] or it could be extracted from an annotated genome. The next step is to determine the number of reads that are aligned to a gene model or transcript. Depending on the type of reference used (genome or transcriptome) various different methods have been established [31, 32]. Finally, the number of reads assigned to a given gene model or transcript is compared between different conditions to identify differentially expressed genes.

The steps outlined above for a general RNA-seq experiment are suitable to compare gene expression levels between different conditions, stages or tissues of the same species. However, comparison of gene expression between different species or populations of the same species needs to account for differences in gene sequences. In this case, reads should be mapped to species-specific references for which the expression level of a gene in one of the species is compared to the expression level of its ortholog in the other species. Most importantly, this requires sets of orthologous genes reliably identified in all references. Since genomes or transcriptomes are usually generated by different research groups for different applications and using different pipelines for assembly and annotation, annotated references for inter-specific gene expression studies are often not comparable. For instance, orthologous genes might be missing from one or more of the references as result of natural variation or technical problems like incomplete assemblies or too many sequencing errors, which hampers unequivocal identification of orthologous genes. Additionally, it is common practice to filter out genes that are incomplete or lack synteny in relation to a model reference from new gene model predictions [33]. Even though there are many tools available to perform genome annotation, a general standard does not exist. Therefore, the final gene set generated by each genome project will have genes missing as a result of methodological problems and filtering criteria, and this can directly influence the result of the differential gene expression analysis [34].

Additionally, even if most one-to-one orthologs have been successfully identified in different references, these gene models may vary in length for various reasons: First, the genes could naturally differ in length among species. Second, as a consequence of differences in the sequence or assembly quality of the reference genomes (e.g. stretches of Ns or premature stop codons due to sequencing errors, incorrect scaffolding or repetitive regions), orthologous gene models might be truncated in one or more of the references. To our knowledge, a comprehensive evaluation of methods that could be applied to account for inter-specific gene length differences has not been performed yet.

A plethora of statistical approaches have been developed to determine whether differences in the number of reads are due to technical variation or due to real biological differences in gene expression. Detailed evaluation and comparison of these methods concluded that the most accurate statistical validation of differential gene expression is reached when statistical models are used that directly take the number of aligned reads into account [35–38]. These methods include standard and generalized Poisson and negative binomial distributions to model count-based expression data [38, 39] as implemented in DESeq [40], DESeq2 [41], edgeR [42] or deGPS [39]. Also the differential expression analysis based on moderated t-statistics as implemented in the limma package [43, 44] using log-transformed count per million values originating from normalization with voom [45] (referred to as voom-limma below) has been shown to perform extremely well [37]. While all of these methods account for most technical biases and control well false positive rates, none of these methods is specifically designed to account for gene length differences as they occur in inter-specific expression studies. One potential solution could be the application of the normalization method reads per kilobase per million mapped reads (RPKM) as it accounts for length differences in gene models [46]. However, it has been shown that even after correcting for length differences, a longer transcript is more likely to appear as differentially expressed if RPKM values are used to assess the statistical significance [35, 37, 47–49]. RPKM normalization is still widely used to compare gene expression levels of different genes within a species, but to our knowledge it has not been tested if this method efficiently normalizes length differences when comparing gene expression in different species.

Here we show that the published genomes of three closely related *Drosophila* species, *D. melanogaster, D. simulans* and *D. mauritiana* have qualitative limitations as references for comparative gene expression studies. This is mainly due to the fact that many genes cannot be properly compared because orthologous genes are missing in the annotation of at least one of the genomes. Even after a direct re-annotation of the three genomes using the same annotation pipeline many orthologous gene models exhibit significant length differences. Taking advantage of these inter-specific gene length differences in the published and the directly re-annotated references, we benchmarked four statistical frameworks (DESeq2 without length correction, DESeq2 with length correction, RPKM-limma and RPKM-voom-limma) for their ability to reduce the number of potentially false positives. We demonstrate that none of these methods sufficiently accounts for the observed differences in gene length. Therefore, we propose that the length normalization should be performed prior to read mapping during the generation of the mapping references. We report a straightforward re-annotation method that relies on a reciprocal re-annotation of orthologous gene models in two or more species. This approach allows the comparison of nearly all genes that have been annotated in *D. melanogaster* in all three species. Additionally, we find that the use of the new gene sets as mapping references results in a more robust estimation of transcript abundance and a more reliable comparison of gene expression levels between species. We propose that the generation and annotation of new genome resources or the re-annotation of existing genomes will be powerful tools to establish gene expression profiling in many emerging model systems.

## Results and discussion

### Analysis of published genome annotations reveals a reduced number of comparable gene models for differential gene expression studies between species

We first assessed the completeness and comparability of the published gene sets for the three closely related species *D. melanogaster*, *D. simulans* and *D. mauritiana*. At the time of our analysis, the annotation of the *D. melanogaster* genome (r5.55) - one of the best curated metazoan genomes available at FlyBase [50–52] - included 13,676 unique protein coding genes. The most recent annotations for *D. simulans* [53] and *D. mauritiana* [54] were generated using the *D. melanogaster* gene set as reference (for the *D. simulans* project the authors used the *D. melanogaster* annotation r5.33, and for the *D. mauritiana* project r5.32 was used). Both gene sets contain a large fraction of the 13,676 *D. melanogaster* genes (86.55 % in *D. simulans* and 87.78 % in *D. mauritiana*, Table 1). However, orthologs of almost 2000 *D. melanogaster* genes are not included in each of the final gene sets either because the authors applied various filtering steps to exclude incomplete orthologous sequence with respect to the *D. melanogaster* gene (see the filtering criteria in the Methods of [53, 54]) or because the genes are not present in one of the species. Since these filtering steps are influenced by the quality of each of the assembled genome and the scientific question of each research group, the missing genes in both annotations are not the same. Only 9994 genes (73.08 %) can be identified unequivocally as orthologs in all three annotations (see Methods). Among the genes missing in at least one annotation, we found some important and well-studied developmental genes including the Hox genes *abdominal B* (*abd-B*), *Ultrabithorax* (*Ubx*) or *Antennapedia* (*Antp*), the head and brain patterning gene *orthodenticle (otd)* and the segment polarity gene *hedgehog* (*hh*) (Additional file 1: Table S1).

**Table 1:** Number of genes obtained by each annotation method

Method	*D. melanogaster*	*D. simulans*	*D. mauritiana*	Comparable
Published annotation	13,676	11,837 (86.55 %)	12,005 (87.78 %)	9,994 (73.08 %)
after filtering				8,810 (64.42 %)
Direct re-annotation	13,676	13,436 (98.24 %)	13,401 (97.99 %)	13,328 (97.45 %)
after filtering				12,334 (90.19 %)
Reciprocal re-annotation	13,457 (98.40 %)	13,373 (97.78 %)	13,346 (97.59 %)	13,311 (97.33 %)
after filtering				13,239 (96.80 %)

The last column contains the number of genes for which 1:1 orthologs were identified in the three species. “after filtering” indicates the remaining common genes after filtering out genes with length difference larger than 49 bp. Percentages in brackets are always given in relation to the total number of gene models in *D. melanogaster* (r5.55; 13,676 gene models)

**Fig. 1:**
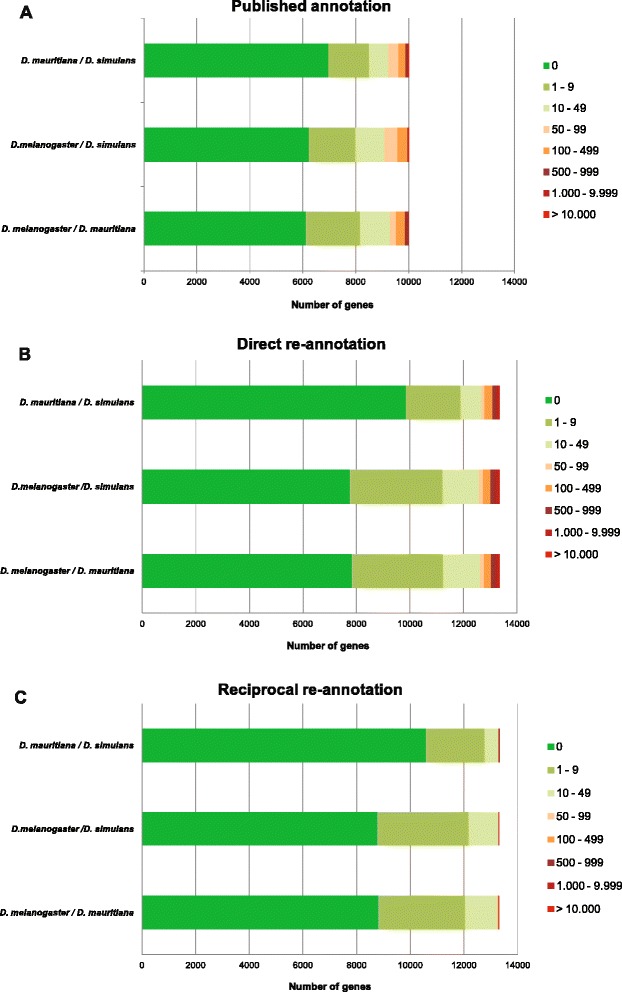
Pairwise length difference between orthologous genes. Bars indicate the number of genes with that difference in length (calculated in number of nucleotides in the annotated transcripts) for each pair of species. Green shades indicate differences lower than 50 bp while orange to red indicate larger differences. The comparison is showed for (**a**) the published annotations, **b** the direct re-annotation of the published genomes and **c** the reciprocal re-annotation of the published genomes

Next we assessed the comparability of the three reference genome annotations in terms of gene length, since length differences larger than the length of the RNA-seq reads are likely to introduce a bias during mapping and the subsequent differential expression analysis. If we consider 50 bp single-end reads, which have been shown to be long enough to produce accurate results when measuring differential gene expression [55–57], genes that have a length difference larger than 49 bp among the annotations of the three *Drosophila* species are likely to bias a subsequent differential gene expression analysis. A pairwise comparison of annotated gene length for the 9994 genes present in all three *Drosophila* species (Fig. 1a) shows that in the published annotations, the gene length differences are larger than 49 bp in 7.6 % (757) of the orthologous genes between *D. mauritiana* and *D. simulans*, 9.1 % (912) between *D. melanogaster* and *D. simulans* and 7.1 % (706) between *D. melanogaster* and *D. mauritiana* (Fig. 1a, Additional file 2: Table S2). If these length differences are not accounted for, these genes could result in false positives in a differential gene expression analysis.

### Direct re-annotation of published genomes

Next we asked whether a direct re-annotation of the *D. simulans* and *D. mauritiana* genomes individually using the same *D. melanogaster* gene set as reference and the same annotation pipeline allows the comparison of more genes in an inter-specific differential gene expression study.

We used the 13,676 protein sequences of *D. melanogaster* (r.5.55) as reference to re-annotate the published genomes of *D. simulans* [53] and *D. mauritiana* [54] using the program Exonerate [58]. Without applying any filtering, we find orthologs of 13,328 *D. melanogaster* genes that are comparable among the two species (Table 1). Next, we determined the length of directly re-annotated genes that are found in all three species. This comparison shows an increase in number of genes with the same length between the three species after direct re-annotation (Fig. 1b; Additional file 2: Table S2). However, a high number of orthologous genes have a length difference of more than 49 bp (Fig. 1b; Additional file 2: Table S2): 706 (5.3 %) between *D. melanogaster* and *D. mauritiana*, 740 (5.6 %) between *D. melanogaster* and *D. simulans* and 658 (4.9 %) between *D. mauritiana* and *D. simulans*. These observed length differences could be due to real natural variation in coding sequence length between species or they could be technical artifacts, for example truncated gene sequences arising from sequencing or genome assembly errors.

In summary, although annotated genomes are available for the three closely related *Drosophila* species *D. melanogaster, D. simulans* and *D. mauritiana*, their use as mapping references for inter-species differential gene expression analyses is limited due to missing orthologs and a potential bias because of different annotated gene lengths. The use of the same reference gene set, annotation pipeline and the lack of filtering incomplete gene sequences results in an increase in the number of comparable genes in these three closely related species.

### Length difference in reference genes introduces biases in differential expression studies

Since we find a high number of gene models with length differences > 49 bp in the published annotations and after the direct re-annotation (Fig. 1a and b; Additional file 2: Table S2), the three *Drosophila* genomes are excellent models to test whether length differences larger than the read length do indeed influence the statistical analysis of differential gene expression. We used the published *D. melanogaster* annotation and the newly generated direct re-annotations of *D. simulans* and *D. mauritiana* as mapping references for pairwise comparisons of gene expression between *D. melanogaster* and *D. mauritiana* and *D. simulans* and *D. mauritiana* using 50 bp single-end Illumina RNA-seq reads generated for these three species (Torres-Oliva et al., in preparation; see Methods). RNA-seq reads generated from one species were mapped only to the gene set of that species.

Using this experimental setup, we compared four different statistical frameworks, namely DESeq2, DESeq2 with gene length correction [41], limma with length correction based on RPKM [43, 44, 46, 59] and voom-limma [45] including RPKM length correction. For each method, we first report the number of differentially expressed genes for each of the two pairwise species comparisons. Next we evaluate the impact of the length differences between gene models on the fold-change in gene expression between species. And eventually, we compare the results of each of the four methods to an independent qPCR experiment for a subset of genes.

#### DESeq2 without length correction

First we performed the statistical analysis for differential gene expression with DESeq2 [41] using directly the read counts for each gene model. For both pairwise comparisons using the published and the direct re-annotation as reference, we found that between 19.9 and 29.7 % of all comparable genes are significantly differentially expressed (Table 2).

**Fig. 2:**
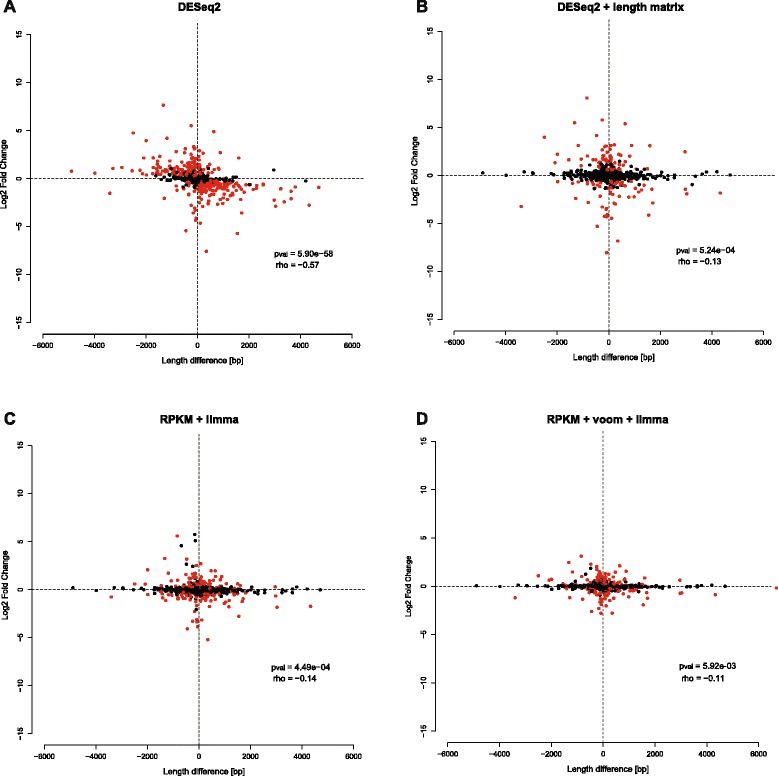
Length differences between orthologous genes introduce gene expression biases. Relation between length differences and the log2-fold change in the RNA-seq experiment between *D. mauritiana* and *D. simulans* using the direct re-annotation of these species as mapping references. Dots represent genes with length difference > 49 bp in these annotations (658 genes). Genes significantly differentially expressed in the presented analysis (*padj* < 0.05) are shown in red. A negative log2-fold change indicates higher expression in *D. mauritiana.* A positive length difference indicates that the ortholog of *D. mauritiana* is longer. The *p*-value and rho of the Spearman’s rank correlation are indicated on the lower right side of the plots. **a** Results of DESeq2 without length correction. **b** Results of DESeq2 applying length normalization factor matrix. **c** Results of applying RPKM normalization and limma to call differentially expressed genes. **d** Results of applying voom normalization followed by a length normalization matrix and limma to call differentially expressed genes

**Fig. 3:**
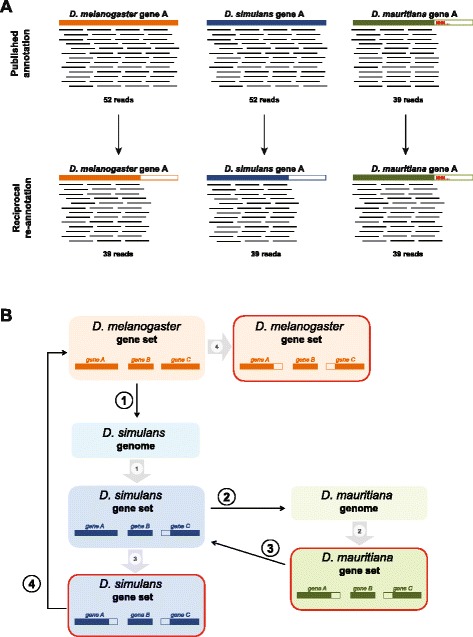
Schematic representation of length bias in inter-species differential expression analysis and our reciprocal re-annotation strategy to correct it. **a** Length bias in the analysis of a non-differentially expressed gene. Coloured rectangles represent the part of the transcript which is included as reference for the RNA-seq reads to map to, while unfilled rectangles are regions of the transcript which are omitted and to which RNA-seq reads cannot be mapped. Red “N”s represent sequencing errors that prevent the complete annotation of a transcript. Mapped reads are shown as thin black lines and the number bellow indicates the total of reads mapped. (*upper panel*) If one transcript is shorter in one of the references compared to its orthologs, for the same expression levels fewer reads will map to it. This can result in false positives in the analysis of differential expression. (*lower panel*) Our strategy to correct this bias is to shorten the orthologs in the other references to match the length of the shorter sequence. **b** Pipeline of reciprocal transcriptome re-annotation method. Black numbers in white circles represent genome annotation steps using the “est2genome” command of Exonerate [58]. Grey numbers in grey circles represent conversion of the resulting GFF file into a new transcript set. Filled horizontal bars represent the annotated set of transcripts; non-filled horizontal bars at the start/end of the transcripts represent parts of the transcript that cannot be correctly annotated in one reference and are therefore eliminated from the transcript set. The boxes with red frame indicate the transcript sets that will be used as reference for RNA-seq read mapping (after confirmation by reciprocal blast). *Step 1*: the transcript set of the best annotated genomes (*D. melanogaster* in our study) is used to annotate one of the other genomes (*D. simulans* in our study) and generate a new transcript set for this species. Due to sequencing errors, some transcripts will be shorter. *Step 2*: the new transcript set form *D. simulans* is used to annotate the last genome (*D. mauritiana* in our study). The gene set generated contains shorter transcripts due to sequencing errors in *D. mauritiana* but also in *D. simulans. Step 3*: the transcript set from *D. mauritiana* is used to re-annotate the previously generated set from *D. simulans* to integrate the information from the *D. mauritiana* assembly. *Step 4*: the second transcript set from *D. simulans* is used to annotate the *D. melanogaster* set in order to integrate the information from *D. simulans* and *D. mauritiana*

Additionally, we found a very strong correlation between inter-specific length differences of the gene models (considering only those gene models with differences > 49 bp) and the log2-fold change in gene expression (Fig. 2a, Additional file 3: Figure S1; Table 2). The negative correlation means that genes that are longer in one species appear to be more up-regulated and vice versa. The correlation can be explained by the mapping procedure: in orthologous genes with different length, more reads align to the ortholog that has the longer gene model (Fig. 3a, upper panel). This results in an artificially higher expression for this specific gene in the species with the longer gene model. From this correlation we also see that most of those genes with length differences and a high log2-fold change are also significantly differentially expressed (Fig. 2a, Additional file 3: Figure S1, *p* < 0.05, red dots), showing that this method introduces a large number of false positives.

**Fig. 4:**
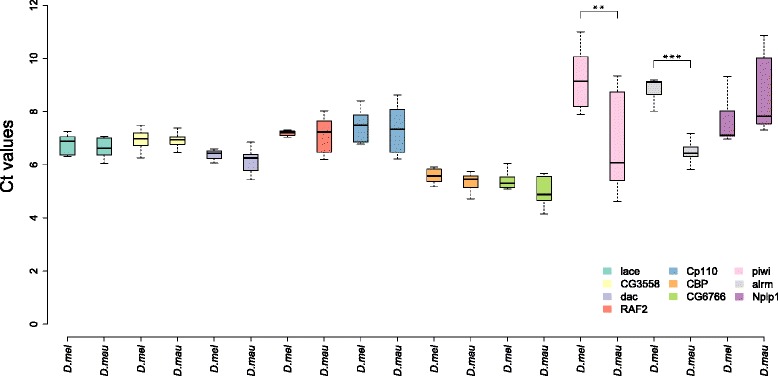
*qPCR results*. Boxplot of normalized Ct values (reference gene: *actin 79B*) For each studied gene (one colour) boxplot is showed for Ct values in *D. melanogaster* OreR (“D. mel”) and *D. mauritiana* TAM16 (“D. mau”). (Significance calculated by t-test (for genes with homogeneous distribution of variances) or t-Welch-test (for genes with not homogeneous distribution of variances); *p < 0.05, **p < 0.005; ***p < 0.0005)

In order to specifically test whether differences in the length of gene models indeed influence the differential expression analysis we chose seven genes that were shorter in the *D. mauritiana* published annotation compared to the published *D. melanogaster* annotation. When we analysed the differential expression using DESeq2 without any length correction, the expression of all seven genes were significantly different (Table 3). The log2-fold change value indicated that *D. melanogaster* had a significantly higher expression than *D. mauritiana* (Table 3). To validate the results obtained by the RNA-seq experiment, we measured the relative expression of the seven genes in *D. melanogaster* and *D. mauritiana* using qPCR. This analysis showed that the seven genes that had length differences in the species-specific annotations were not significantly differentially expressed (Fig. 4, Table 3). As a control we chose another three genes that showed significant differential expression in the RNA-seq data but had the same length in both species in the two annotation methods (*piwi* and *alrm* are significantly higher in *D. mauritiana* and *Nplp1* is higher in *D. melanogaster*). We found that *piwi* and *alrm* showed a significantly higher expression in *D. mauritiana* when using this alternative quantification method*,* confirming the results obtained by RNA-seq (Fig. 4, Table 3). Moreover, *Nplp1* had higher expression in *D. melanogaster* again consistent with our RNA-seq data, although this difference was not significant (*p* = 0.072; Fig. 4, Table 3).

**Table 2:** Differentially expressed genes and correlation between calculated log2-fold changes and length difference between orthologous genes. Results are shown for the four applied methods, the three studied annotation strategies and the two described pairwise species comparisons

Method	Annotation	Species	# Common genes	# Differentially expressed genes (% of common genes)^a^	Spearman’s *p* value^b^	FDR 0.05	Spearman’s rho^b^
DESeq2	Published	*D. mau* vs *D. mel*	11,503	2,438 (21.2)	6.52e-33	***	−0.36
		*D. mau* vs *D. sim*	10,023	2,974(29.7)	2.15e-20	***	−0.33
	Direct	*D. mau* vs *D. mel*	13,401	2,665 (19.9)	3.35e-20	***	−0.33
		*D. mau* vs *D. sim*	13,328	3,710 (27.8)	5.90e-58	***	−0.57
	Reciprocal	*D. mau* vs *D. mel*	13,331	2,501 (18.8)	5.12e-02	n.s.	−0.23
		*D. mau* vs *D. sim*	13,320	3,508 (26.3)	1.48e-01	n.s.	−0.29
DESeq2 + length matrix	Published	*D. mau* vs *D. mel*	11,503	1,192 (10.4)	2.24e-05	***	−0.13
		*D. mau* vs *D. sim*	10,023	1,545 (15.4)	3.20e-02	^a^	−0.08
	Direct	*D. mau* vs *D. mel*	13,401	1,259 (9.4)	1.07e-02	^a^	−0.09
		*D. mau* vs *D. sim*	13,328	1,957 (14.7)	5.24e-04	**	−0.13
	Reciprocal	*D. mau* vs *D. mel*	13,331	1,215 (9.1)	7.03e-01	n.s.	−4.6e-02
		*D. mau* vs *D. sim*	13,320	1,910 (14.3)	7.34e-01	n.s.	−0.07
RPKM + limma	Published	*D. mau* vs *D. mel*	11,503	1,904 (16.6)	4.42e-04	***	−0.11
		*D. mau* vs *D. sim*	10,023	2,427 (24.2)	1.06e-03	**	−0.12
	Direct	*D. mau* vs *D. mel*	13,401	1,890 (14.1)	5.68e-03	^a^	−0.10
		*D. mau* vs *D. sim*	13,328	2,795 (21,0)	4.49e-04	***	−0.14
	Reciprocal	*D. mau* vs *D. mel*	13,331	1,830 (13.7)	5.92e-01	n.s.	−6.4e-02
		*D. mau* vs *D. sim*	13,320	2,738 (20.6)	2.83e-01	n.s.	−0.22
RPKM + voom + limma	Published	*D. mau* vs *D. mel*	11,503	1,853(16.1)	9.39e-04	***	−0.10
		*D. mau* vs *D. sim*	10,023	2,204(22.0)	4.63e-02	^a^	−0.07
	Direct	*D. mau* vs *D. mel*	13,401	1,899(14.2)	1.01e-02	^a^	−0.10
		*D. mau* vs *D. sim*	13,328	2,607(19.6)	5.92e-03	^a^	−0.11
	Reciprocal	*D. mau* vs *D. mel*	13,331	1,819(13.6)	5.79e-01	n.s.	−0.07
		*D. mau* vs *D. sim*	13,320	2,519(18.9)	4.06e-01	n.s.	−0.17

^a^FDR 0.05

^b^Spearman’s rank correlation is measured between log2FC and length difference of genes showing more than 49 bp length difference

Published annotation: D. mau vs. D.mel: 1,038 genes/D.mau vs. D.sim: 764 genes; Direct annotation: D.mau vs. D.mel: 716 genes/D.mau vs. D.sim: 658 genes; Reciprocal annotation: D.mau vs. D.mel: 71 genes/D.mau vs. D.sim: 26 genes

*p < 0.05; **p < 0.005; ***p < 0.0005

In summary, these results suggest a high level of potentially false positive candidates when methods based on direct read counts without the application of length correction are used with mapping references that exhibit differences in the length of orthologous genes.

#### DESeq2 with length correction

Next we benchmarked the use of DESeq2 [41] including a normalization factor matrix incorporating gene length to account for the length differences between orthologous genes. Using this approach for pairwise gene expression analyses, we found that only 9.4 to 15.4 % of the comparable genes were significantly differentially expressed. Even though the gene length was accounted for during the DESeq2 analysis of differential gene expression, we still find a correlation between inter-specific gene length differences and log2-fold changes (Fig. 2b, Additional file 4: Figure S2, Table 2). However, the significance of this correlation is greatly reduced in comparison to the DESeq2 analysis without length correction (Table 2), suggesting that the length correction incorporated in DESeq2 helps to reduce the number of false positive candidate genes. This finding is further supported by the comparison of the RNA-seq results to the qPCR data. After length correction only one (*Cp110*) of the seven genes that are longer in *D. melanogaster* show significant differential expression (Fig. 4, Table 3).

#### limma with RPKM length correction

RPKM values are commonly calculated for RNA-seq datasets to account for variation in library sizes and to correct for length differences between different genes within the same species [46]. The moderated t-statistics incorporated in the limma R package [43, 44] can subsequently be used to assess differential gene expression. It has not been tested if this approach also corrects properly for differences in the length of the same gene being compared between two species. Using this method, we found between 14.1 % and to 24.1 % of the comparable genes to be significantly differentially expressed (Table 2). Our correlation analysis shows that the correction of a length bias using RPKM values still results in a clearly significant correlation between the gene length difference and the observed log2-fold change (Fig. 2c, Additional file 5: Figure S3, Table 2). However, compared to the DESeq2 analysis without length correction, the significance values are highly reduced (Table 2), showing that the number of false positives is lower. Accordingly, six of the seven genes that we benchmarked with qPCR show no significant differential gene expression although they show clear length differences between *D. melanogaster* and *D. mauritiana* (Table 3). Again *Cp110* is the only gene that appears as significantly differentially expressed also after correcting for length differences.

#### voom-limma with RPKM length correction

It has recently been shown that differential gene expression analysis with limma [43, 44] using normalized read counts from voom [45] perform very well for RNA-seq datasets [37]. Although this method is designed to work with direct read counts, in this case we tested it with an additional transcript length correction. Between 15 and 23.5 % of the comparable genes are significantly differentially expressed (Table 2). After length correction (RPKM) and normalization with voom, we found a significant correlation between gene length differences and log2-fold changes when the published annotations and the directly re-annotated reference gene sets were used However, this was slightly reduced compared to the RPKM-limma analysis, especially for the *D. simulans* and *D. mauritiana* comparison. (Figure 2d, Additional file 6: Figure S4, Table 2). For the seven qPCR benchmarked genes that have clear length differences between *D. melanogaster* and *D. mauritiana* the use of the voom-limma method results in three significantly differentially expressed genes (Table 3), suggesting a higher false positive rate.

#### Length correction during the statistical analysis might be insufficient

The comprehensive comparison of four methods for differential gene expression analysis shows that the incorporation of a length correction drastically reduces the number of false positive candidate genes. Although the correlation between gene length differences and the observed log2-fold changes (Fig. 2, Additional files 3, 4, 5 and 6: Figure S1-S4, Table 2) is reduced in the three methods that account for gene length differences (length matrix in DESeq2, RPKM-limma and RPKM-voom-limma), none of them sufficiently corrects the length bias present in the two gene sets used as mapping references. This is also supported by the qPCR validation of seven genes that exhibit clear length differences between the published *D. melanogaster* and *D. mauritiana* annotations (Fig. 4, Table 3). In all three methods at least one gene was still artificially significantly differentially expressed. This is most pronounced for the voom-limma method where three of the seven genes are significantly differentially expressed. Of the seven genes we analysed using qPCR, *Cp110* was in all cases identified as a false positive candidate. In order to further characterise this gene, we visually inspected the distribution of mapped reads. Interestingly, the 3′-region is missing in the *D. mauritiana* ortholog of *Cp110* (Additional file 7: Figure S5A) and we found clearly more *D. melanogaster* reads that map to this 3′-part of the transcript than to the 5′-region (Additional file 7: Figure S5C). The number of *D. melanogaster* and *D. mauritiana* reads mapped to the 5′-part of the transcript is comparable (compare Additional file 7: Figure S5B to C). Hence, a very likely explanation for the inefficient length correction of the three applied methods could be an unequal distribution of the mapped reads along the transcripts.

Besides an insufficient length correction, the DESeq2 method including a length matrix results in the lowest number of significantly differentially expressed genes, suggesting that the length correction applied here might be extremely conservative and could lead to a high rate of false negatives. Interestingly, in many pairwise comparisons we found more significantly differentially expressed genes with a higher expression in *D. mauritiana* compared to *D. melanogaster* and *D. simulans* (not shown), although *D. mauritiana* gene models are generally shorter than those of the other two species (Fig. 2, Additional files 3, 4, 5 and 6: Figure S1-S4). This finding suggests that the length correction applied here might reduce the power to detect differential expression for the already short *D. mauritiana* genes.

In summary, all three methods that include a length correction decrease the chance of identifying false positives. The RPKM-voom-limma and RPKM-limma methods seem to give the best ratio of false positives and false negatives, while DESeq2 including a length matrix is very conservative. However, none of the length correction methods tested does efficiently account for all differences in gene length observed in the reference annotation of the three studied *Drosophila* species. The length bias is most obvious when the distribution of reads is not uniform across the transcript body (e.g. *Cp110*). Therefore, all genes that exhibit length differences larger than the read length should be excluded from any of the reference gene sets (see Table 1; number of comparable genes after filtering).

### Reciprocal re-annotation reduces the number of false positive candidates

#### Overview of the reciprocal re-annotation pipeline

To overcome problems due to length differences between orthologous genes and simultaneously maximize the number of comparable genes, we developed a pipeline to reciprocally re-annotate the reference genomes of the three species (Fig. 3b, Methods). Instead of directly annotating the *D. simulans* and *D. mauritiana* genomes individually using the *D. melanogaster* reference gene set, we first annotated the genome of *D. simulans* based on the protein set from *D. melanogaster*. Subsequently, we used these newly annotated *D. simulans* gene models to annotate the genome of *D. mauritiana*. This gene set was then used as a reference to re-annotate again the previously generated *D. simulans* gene set. And finally, we used these *D. simulans* gene models that already contain consensus features of *D. simulans* and *D. mauritiana* to re-annotate the *D. melanogaster* gene set (Fig. 3b). Therefore, we obtained the longest sequence present in all three species and then, if necessary, reduce its length in the other references accordingly. Thus, we expect to equalize the length of all the genes for the three references (Fig. 3a, lower panel). It is important to note here that it does not matter in which order the reciprocal re-annotation is done. As long as the first reference is the one of *D. melanogaster* (i.e. the best curated annotation), we obtained the same results when we first annotate *D. simulans* or *D. mauritiana* (not shown).

#### Reciprocal re-annotation efficiently reduces gene length differences between species

With the reciprocal re-annotation of the published genomes we obtained 97.33 % of the 13,676 *D. melanogaster* gene models in each of the three species (Table 1). In accordance with our expectations, only a small fraction of those genes found in all three species have a length difference of more than 49 bp (Fig. 1c; Additional file 2: Table S2): 71 genes (0.53 %) genes between *D. melanogaster* and *D. mauritiana*, 41 genes (0.3 %) between *D. melanogaster* and *D. simulans* and only 26 genes (0.19 %) between *D. mauritiana* and *D. simulans*. Hence, the reciprocal re-annotation of the published genomes allows the analysis of the highest number of comparable genes with less than 50 bp length differences in a differential gene expression study (Table 1; 13,239 (96.80 %) of the 13,676 *D. melanogaster* gene models).

#### Evaluation of the reciprocal re-annotation in RNA-seq experiments

To quantitatively test whether the number of false positives due to gene length differences is indeed reduced after reciprocal re-annotation, we applied a pairwise analysis of differential gene expression between *D. melanogaster* and *D. mauritiana* and *D. simulans* and *D. mauritiana* (see Methods). We mapped the RNA-seq reads to the new references and performed the statistical analysis using the four methods evaluated above: DESeq2, DESeq2 with length correction [41], RPKM-limma [43, 44, 46], RPKM-voom-limma [45].

As for the published and directly re-annotated references, the statistical analysis with DESeq2 resulted in the highest number of significantly differentially expressed genes (18.8 and 26.3 % of the comparable genes; Table 2). This number clearly dropped to 9.1 and 14.3 % after including a length correction during the DESeq2 analysis. Similarly, the number of differentially expressed genes is greatly reduced if RPKM-limma and RPKM-voom-limma are being used (Table 2). However, only 71 (*D. melanogaster* vs. *D. mauritiana*) and 26 (*D. simulans* vs. *D. mauritiana*) pairwise comparable genes exhibit length differences greater than 49 bp after reciprocal re-annotation. One would expect that only those genes should be affected by any of the three length correction methods.

Therefore, we propose that the combination of a reciprocal re-annotation in combination with a read-count based DESeq2 analysis of differential gene expression is likely to provide the most comprehensive and reliable estimation of inter-specific gene expression differences. This is further supported by the lack of a significant correlation between log2-fold changes and gene length differences if the DESeq2 is used in combination with the reciprocal re-annotation as mapping reference (Additional files 3, 4, 5 and 6: Figure S1-S4; Table 2). Although the correlation is not significant, we still find that most significantly differentially expressed genes with length differences larger than 49 bp have higher expression in the species with the longer transcript (Additional files 3, 4, 5 and 6: Figure S1-S4). Therefore, we propose that those genes should be filtered out from further differential gene expression analysis. Additionally, the seven genes that were validated using qPCR did not show a significant differential expression after their length was equalized (Fig. 4, Table 3), suggesting that the length correction during the annotation of genomes can facilitate the reduction in false positive candidate genes in RNA-seq analyses.

#### Assessment of power to detect differential gene expression

It is important to note that the gene models generated by our reciprocal re-annotation pipeline do not necessarily represent the complete gene and thus the most comprehensive annotation for each species. This is due to the fact that potential full gene models in one species might have been adjusted to the shortest orthologous gene model. Therefore, in each round of annotation some gene models are truncated to fit the length of its orthologs (see Figs. 2 and 3a). If the gene models would be extremely shortened, this could of course lead to a loss of statistical power for the differential gene expression analysis. In order to estimate how much sequence information we really lose, we compared the length of the *D. melanogaster* gene models before and after the reciprocal re-annotation. This comparison shows that 12,642 (92.44 %) of the 13,676 gene models still contain 90 to 100 % of their original sequence length after the reciprocal re-annotation (Additional file 8: Figure S6).

Next we assessed the potential loss of power by comparing the number of mapped reads between the published annotations (13,676 genes in *D. melanogaster*, 12,005 genes in *D. mauritiana* and 11,837 genes in *D. simulans*; Table 1) and the gene sets generated from our reciprocal re-annotation of the published genomes (13,457 genes in *D. melanogaster*, 13,373 genes in *D. simulans* and 13,346 genes in *D. mauritiana*, Table 1). Overall, the proportion of successfully mapped reads for all reference gene sets was between 40 and 67 % (Table 4). A large portion of this relatively low mapping rate can be explained by the fact that we excluded UTR sequences from all reference gene sets, what accounts for about 27.4 % of all mapped reads (see Methods; Additional file 9: Table S3). Additionally, we only used the longest isoform of *D. melanogaster* for all annotations in the other two species (see Methods). Therefore, some differentially spliced exons might not be represented in the newly generated gene sets. However, the use of the sum of all exons only increases the mapping success by 0.4 % if UTRs are excluded and 1.6 % if the UTRs are included (Methods; Additional file 9: Table S3). If the comparison of the expression of different isoforms across species is of interest one could perform the quantification on the level individual transcripts [60] or even exons. This approach requires of course a proper annotation of the different isoforms in all reference genomes and a dedicated mapping pipeline. For our analysis, we found for all replicates more than 17 million mapped reads after reciprocal re-annotation (Table 4) what has been shown to provide enough statistical power for differential gene expression analyses [61].

**Table 3:** Analysis of differential expression

		Published transcriptomes						Reciprocally re-annotated transcriptomes		
Gene	qPCR	Gene length (# nucl.)		DESeq2	DESeq2 + length matrix	RPKM + limma	RPKM + voom + limma	Gene length (# nucl.)		DESeq2
	*log2FC*	*D. mel*	*D. mau*	*log2FC*	*log2FC*	*log2FC*	*log2FC*	*D. mel*	*D. mau*	*log2FC*
lace	−0.19	1791	903	1.40***	0.38	0.41	0.15	902	902	0.03
CG3558	0.08	3147	1956	1.50***	0.67	0.75	0.26*	3150	3135	0.16
dac	−0.29	3243	1878	1.47***	0.57	0.65	0.18	1887	1878	0.46
RAF2	1.0e-03	3351	1854	1.77***	0.84	0.94	0.31	1959	1966	0.33
Cp110	−0.18	1998	1218	2.31***	1.38*	1.4**	0.55**	2000	1998	0.11
CBP	−0.21	1653	894	1.42***	0.35	0.54	0.14	1656	1653	−0.24
CG6766	−0.41	1575	852	1.81***	0.79	0.88	0.25*	855	855	0.31
piwi	−2.60**	2529	2526	−2.48***	−2.54***	−1.99**	−1.08**	2532	2529	−2.48***
alrm	−2.37***	1413	1413	−6.54***	−6.67***	−4.93***	−2.68***	1416	1416	−6.49***
Nplp1	1.04	1461	1461	3.85***	3.63***	3.06***	1.50***	1464	1464	3.80***

Expression comparison is for *D. mauritiana* vs. *D. melanogaster*, thus a positive log2-fold change (log2FC) indicates higher expression in *D. melanogaster* and vice versa. *p < 0.05; **p < 0.005; ***p < 0.0005

**Table 4:** **|** List of RNA-seq samples and the percentage and number of mapped reads to different reference transcriptomes

Sample	Original read type^a^	Published transcriptomes		Reciprocally re-annotated transcriptomes	
		Percentage	Total number of mapped reads	Percentage	Total number of mapped reads
*D. melanogaster* replicate A	SE 50 bp	58.86 %	28,486,024	57.33 %	27,744,730
*D. melanogaster* replicate B	SE 50 bp	44.23 %	17,675,472	43.19 %	17,260,775
*D. melanogaster* replicate C	SE 50 bp	65.51 %	25,316,846	63.91 %	24,699,746
*D. mauritiana* replicate A	SE 50 bp	40.70 %	16,575,011	43.31 %	17,639,874
*D. mauritiana* replicate B	SE 50 bp	56.17 %	31,884,442	60.07 %	34,100,435
*D. mauritiana* replicate C	SE 50 bp	53.01 %	23,653,723	56.98 %	25,425,486
*D. mauritiana* replicate D	PE 100 bp	56.06 %	111,643,922	61.07 %	121,610,905
*D. mauritiana* replicate E	PE 100 bp	54.28 %	130,638,956	59.51 %	143,226,939
*D. mauritiana* replicate F	PE 100 bp	60.90 %	144,541,354	66.21 %	157,165,639
*D. simulans* replicate A	PE 100 bp	62.26 %	118,272,529	66.71 %	126,741,807
*D. simulans* replicate B	PE 100 bp	57.90 %	138,364,665	62.56 %	149,508,494
*D. simulans* replicate C	PE 100 bp	56.32 %	150,692,651	60.98 %	163,168,587

^a^ Reads originally 100 bp paired-end (PE) were split in half to be 50 bp each and treated as single-end (SE) reads

We observed an increase in the mapping percentage of up to 5 % in *D. simulans* and *D. mauritiana* when the reciprocally re-annotated gene sets are used as references (Table 4). This result shows that, although some gene models were now shorter, many genes that had been filtered out in the published genome annotations are actually expressed in these species. The use of the re-annotated gene set only slightly decreases the mapping success by 1 to 1.6 % in *D. melanogaster* (Table 4), which is likely to be due to the artificial shortening of *D. melanogaster* gene models.

In summary, we show that the artificial shortening of transcripts after reciprocal re-annotation does not have a major impact on the power to detect differential gene expression.

#### Practical considerations

We demonstrate that the use of all annotated exons instead of the longest isoform of each gene model does not significantly increase the power to detect differential gene expression. In contrast, the inclusion of UTR regions for the reciprocal re-annotation will clearly increase the number of mapped reads (Additional file 9: Table S3) and hence the statistical power. However, the availability of UTR sequence information strongly depends on the quality of the annotation of the species to compare, since UTR and isoform predictions usually profit from the presence of RNA-seq data to be incorporated in the annotation pipeline. Additionally, the annotation of UTR regions might become more complicated if more distantly related species are studied, because UTR regions tend to evolve faster than coding region [62].

Although we used very closely related species for our analysis here, we think that the presented reciprocal re-annotation is also applicable for genomes of more distantly related species. As a consequence of a higher sequence divergence between distantly related species, inter-specific gene length differences are likely to be more pronounced. If such genomes were used as mapping references, the direct use of length correction during the statistical analysis of differential gene expression might enhance the over-correction effect that we have demonstrated for the three presented methods. Additionally, if the gene lengths are very different between species, the length bias that has been reported for RPKM based normalization approaches [35, 37, 47–49] might be more pronounced. Therefore, we propose that the correction of the inter-specific length bias prior to read mapping using our reciprocal re-annotation pipeline should result in more robust results. However, for more distantly related species, the reciprocal re-annotation is likely to result in more artificial shortening of the genes. Since this could reduce the power to detect differential gene expression, we propose to assess the length differences between species as we presented it here (Fig. 1) prior to the sample preparation and sequencing and to adjust the coverage accordingly by generating more reads to increase sequencing depth.

In the presented case, at least one of the three *Drosophila* species represents a well-established model system with a high quality genome assembly and annotation. If this is available, the reciprocal re-annotation pipeline should of course be started with the highest quality annotation. If the annotation quality of all genomes similar the pipeline could be started with any of the studied species, since we showed that the direction of the reciprocal annotation does not influence the final result. However, if the quality of all annotations is comparably low, one should consider generating longer paired-end reads with higher coverage to first perform a *de novo* annotation with tools like AUGUSTUS [63, 64] or BRAKER1 [65] using those reads to train the respective algorithm. Subsequently, the generated RNA-seq reads can be used to assess differential gene expression using the reciprocally re-annotated references with length adjusted orthologous genes.

## Conclusions

We have carried out a comprehensive comparison of the annotations of published genomes for the three closely related *Drosophila* species, *D. melanogaster, D. simulans* and *D. mauritiana*. This analysis reveals that different assembly strategies, annotation pipelines and filtering steps result in only a small fraction of genes that are comparable among all three species. A direct re-annotation of the *D. simulans* and *D. mauritiana* genomes using the same *D. melanogaster* reference gene set and the same annotation pipeline significantly improves the comparability of the gene sets. However, this direct re-annotation still results in length differences in many gene models between species. Based on these length differences between orthologous genes we tested four alternative methods to statistically assess differential gene expression using RNA-seq, namely DESeq2, DESeq2 with length correction, RPKM-limma and RPKM-voom-limma. We show that none of these methods sufficiently accounts for the inter-specific gene length differences what is evident by a high number of false positive differentially expressed genes. This finding is further supported by qPCR as an alternative transcript quantification method.

In order to further reduce the observed false positive rate, we argue that the length bias should be accounted for prior to the RNA-seq analysis during the generation of the mapping references. Therefore, we implemented a robust reciprocal re-annotation pipeline that allows the generation of highly comparable gene sets to serve as mapping references for inter-specific RNA-seq experiments. Applying RNA-seq and qPCR we confirm the successful reduction of false positive candidate genes if the reciprocally re-annotated genomes are used as mapping references. The reciprocal re-annotation pipeline can easily be adopted to re-annotate genomes of other closely related species or populations of animals and plants. Although we introduced our novel approach here to re-annotate three genomes at a time, it can of course be applied to two or more genomes.

## Methods

### Comparison of published annotations

We obtained the complete coding sequence (CDS) set of *D. melanogaster* r5.55 from FlyBase and considered only the longest isoform of each gene. Because identical sequences cannot be distinguished when RNA-seq reads are mapped (e.g. 23 different Histone 3 loci), we only retained one copy of genes with exactly the same nucleotide sequence (49 sequences, 195 transcripts discarded).

The genome and annotation of *D. mauritiana* was downloaded from http://www.popoolation.at/mauritiana_genome/index.html [54], combining the five gene set files. The transcript set was obtained from a GFF file and the *D. mauritiana* genome. IDs were converted with the FlyBase conversion tool.

The genome and annotation of *D. simulans* was downloaded from http://genomics.princeton.edu/AndolfattoLab/w501_genome.html [53], combining “clean” and “unclean” data sets. The transcript set was obtained from a GFF file and the *D. simulans* genome.

Common genes were identified by gene ID (FBgn nomenclature) correspondence in all species. Genes absent from these species-specific annotations were identified by comparing the annotated genes to the genes present in the *D. melanogaster* gene set (Additional file 1: Table S1). The absence of these genes was confirmed by tblastn [66] search.

### Direct re-annotation of genomes

The *D. mauritiana* and *D. simulans* genomes were obtained as described above and annotated with the *D. melanogaster* CDS set using Exonerate v2.2 [58] with the command –-model est2genome --softmasktarget yes --bestn 1 --minintron 20 --maxintron 20000. The resulting GFF files were converted into transcript sets for each species from the corresponding genome files.

For some genes these three species have a different number of paralogs. For differential expression analysis it is essential to only consider orthologs of each gene, i.e. the number of reads that map to one transcript in one species cannot be reliably compared to the number of counts in two or more transcripts in another species. To count the total number of recovered transcripts in each annotation round, we kept only one copy of transcript sequences that gave more than one best hit in the target set. We selected the copy to keep based on conserved synteny (the putative paralog that is in the same chromosome and relative strand in the target genome and that has the same neighbouring genes as in *D. melanogaster*) and conserved gene structure (the putative paralog that has the same number of exons as *D. melanogaster*). Genes for which none of the multiple copies found satisfied these conditions were discarded. In the *D. mauritiana* direct re-annotation only one gene gave more than one predicted copy (FBgn0264343); since none of the copies was in the same chromosome as *D. melanogaster* (2 L) they were discarded. In the *D. simulans* direct re-annotation five genes gave more than one copy (FBgn0002933, FBgn0010294, FBgn0036177, FBgn0053874 and FBgn0062565); for the first three genes, the copy that was in the same relative strand as *D. melanogaster* was kept, FBgn0053874 was discarded because none of the copies was in the same chromosome as *D. melanogaster* (2 L) and for FBgn0062565 only the copy predicted in the same chromosome as *D. melanogaster* (X) and with the same number of exons (3) was kept and the other was discarded.

BLAST 2.2.26+ [66] was used to back-blast the resulting gene sets to the *D. melanogaster* gene set (blastn -max_target_seqs 1). Only the genes that had as best hit the *D. melanogaster* gene that had been used to annotate them (reciprocal best hit) were kept and reported in Table 1.

### Generation of comparable transcriptomes – reciprocal re-annotation pipeline

To generate reference transcriptomes for the three species with a minimum length difference between orthologous sequences and including the maximum number of transcripts present in all species for analysis of inter-specific differential expression, we annotated the transcript sets of the different species via multiple rounds of pairwise alignment with Exonerate v2.2 [58] following the scheme shown in Fig. 3b. Since FlyBase [50] maintains an up to date curation and annotation the of *D. melanogaster* genome, we used this gene set as the first reference.

We used the *D. melanogaster* CDS set (r5.55) to annotate the *D. simulans* reference genome (Fig. 3b, step 1) with exonerate –-model est2genome --softmasktarget yes --bestn 1 --minintron 20 --maxintron 20000. The resulting gene set was used to annotate the *D. mauritiana* reference genome using –-model est2genome (Fig. 3b, step 2). At this point, the transcript set contains the maximized number of comparable genes and minimized transcript length difference between the three species’ references. Consequently, step 3 consisted of reciprocally annotating the *D. simulans* transcript set with the *D. mauritiana* transcript set (Fig. 3b, step 3) and finally using the resulting *D. simulans* transcript set to annotate *D. melanogaster* transcript set (Fig. 3b, step 4). The criteria used to deal with multiple paralogs was the same as described above when the annotation reference was a genome (steps 1 and 2). Step 1 was the same as previously described and only one copy of FBgn0002933, FBgn0010294, FBgn0036177 and FBgn0062565 were kept. In step 2, only one gene (FBgn0263247) gave two hits in *D. mauritiana*; these two were clear tandem duplicates and the one predicted at 3 L:11061688-11061810 was kept. In steps 3 and 4 only the genes where the gene ID of the target and the query matched were kept.

A back-blast to the original *D. melanogaster* gene set was also performed with the resulting gene sets of the three species. Only the reciprocal best hits were kept and reported in Table 1.

A list of gene names (FBgn nomenclature) and the respective transcript lengths for all annotations used in this study (published annotations, direct re-annotation and the reciprocal re-annotation) of all three species are available as part of the processed files uploaded to the Gene Expression Omnibus (GEO) database (Accession number: GSE76252). Additionally, gff and fasta files of the final datasets and of intermediate steps of the reciprocal re-annotation pipeline are available from GSE76252 as well.

### RNA isolation and sequencing

RNA–seq reads for analysis of differential expression were generated for *D. melanogaster* (OregonR), *D. mauritiana* (TAM16, collected in Mauritius in 2007 [54]) and *D. simulans* (*yellow vermillion forked,* YVF; DSSC, University of California, San Diego, Stock no.14021-0251.146) (Torres-Oliva et al., in preparation). In summary, flies were raised at 25 °C and 12 h:12 h dark/light cycle in density‐controlled conditions (30 freshly hatched LI larvae per vial). Female LIII larvae were dissected and eye-antennal imaginal discs were stored in RNALater (Qiagen, Venlo, Netherlands) at 120 h after egg laying. We dissected 40–50 discs per sample and generated three biological replicates for *D. melanogaster* and for *D. simulans* and 6 biological replicates for *D. mauritiana* (total of 12 samples).

Total RNA was isolated using the Trizol (Invitrogen, Thermo Fisher Scientific, Waltham, Massachusetts, USA) method according to the manufacturer’s recommendations and the samples were DNAse I (Sigma, St. Louis, Missouri, USA) treated in order to remove DNA contamination. RNA quality was determined using the Agilent 2100 Bioanalyzer (Agilent Technologies, Santa Clara, CA, USA) microfluidic electrophoresis. Only samples with comparable RNA integrity numbers were selected for sequencing.

Library preparation for RNA-Seq was performed using the TruSeq RNA Sample Preparation Kit (Illumina, catalog ID RS-122-2002) starting from 500 ng of total RNA. Accurate quantitation of cDNA libraries was performed by using the QuantiFluor™dsDNA System (Promega, Madison, Wisconsin, USA). The size range of final cDNA libraries was determined applying the DNA 1000 chip on the Bioanalyzer 2100 from Agilent (280 bp). cDNA libraries were amplified and sequenced by using cBot and HiSeq 2000 (Illumina): single-end reads were generated for *D. mauritiana* (replicates A, B and C) and for *D. melanogaster* samples (1×50 bp) and paired-end reads were generated for *D. mauritiana* (replicates D, E and F) and for *D. simulans* samples (2×100 bp).

Sequence images were transformed to bcl files using the software BaseCaller (Illumina). The bcl files were demultiplexed to fastq files with CASAVA (version 1.8.2). Quality control was carried out using FastQC (version 0.10.1, Babraham Bioinformatics). Only replicates A, D and E from *D. mauritiana* and replicate C from *D. simulans* had bases with Phred quality score < Q20. Following recently published guidelines [67] we did not trim these bases but instead relied on the aligner software to make the quality call. Due to this procedure the overall mapping success (% mapped reads) for all datasets was slightly reduced. Of *D. melanogaster* (replicate A) for example, about 4.8 % of the reads do not map against the entire genome, suggesting that they might be filtered out due to low quality during the mapping procedure (Additional file 9: Table S3).

Raw fastq files of all samples have been deposited in NCBI’s Gene Expression Omnibus [68] and are accessible through GEO Series accession number GSE76252 (http://www.ncbi.nlm.nih.gov/geo/query/acc.cgi?acc=GSE76252).

### Analysis of differential expression

Since we generated two different types of RNA-seq reads (namely 100 bp paired-end and 50 bp single-end), we only compared the datasets that were produced with the same technique, i.e. *D. melanogaster* reads were compared only to *D. mauritiana* 50 bp reads and *D. simulans* reads to *D. mauritiana* 100 bp paired-end reads. Since 50 bp single-end reads are informative enough for differential expression analysis [55–57] and this is the cuttoff we set in our analysis as the maximum gene length difference, prior to mapping, 100 bp paired-end reads from *D. simulans* and *D. mauritiana* were split into two 50 bp reads each. Left and right reads were merged into a single file to be equivalent to single-end reads. 50 bp single-end reads from *D. mauritiana* and *D. melanogaster* were not processed prior to mapping.

Bowtie2 [69] with parameters –very-sensitive-local –N 1 was used in all cases to map the reads to the respective references: *D. melanogaster* reads were mapped to the published gene set (Flybase, r5.55) and to our novel reciprocally re-annotated gene set. *D. mauritiana* and *D. simulans* reads were mapped to the respective published gene sets [53, 54], to the directly re-annotated gene sets and to the reciprocally re-annotated gene sets. The number of reads mapping to each transcript were summarized using samtools v0.1.19 [32].

To calculate the percentage of reads mapped to UTRs we aligned *D. melanogaster* replicate A reads to the longest full transcripts of *D. melanogaster* r5.55 and compared the mapping percentage to that of the mapped reads to the longest CDS set. To calculate the percentage of reads mapped to transcript regions not included in the longest CDS set we aligned *D. melanogaster* replicate A reads to the complete CDS set (including all isoforms) and compared the mapping percentage to that of the longest CDS set. To calculate the percentage of reads generated from unannotated regions we aligned *D. melanogaster* replicate A reads to the complete *D. melanogaster* genome r5.55 and compared the mapping percentage to that of the mapped reads to all annotated transcripts.

Differential expression was determined for each orthologous gene between *D. melanogaster* and *D. mauritiana* (from the originally 50 bp single-end reads) and between *D. simulans* and *D. mauritiana* (from the originally 100 bp paired-end reads). Four different methods were used to call differentially expressed genes for each annotation strategy:

1. DESeq2 [41] (v1.6.3) with direct counts per transcript and default parameters.
2. DESeq2 with a transcript length normalization factor matrix with row-wise geometric means of 1. This matrix was applied with the command normalizationFactors(). The rest of parameters were left as default.
3. Limma [43, 44] (v3.22.7) on reads per kilobase per million (RPKM). RPKM values were calculated for each transcript with the corresponding library size and transcript length. 1 was added to the resulting value to prevent negative values when applying log transformation. Limma was applied to log_2_ transformed RPKM values to call differentially expressed genes using ebayes(trend = T).
4. RPKM-voom-limma [45]. RPKM values were calculated as described above and voom() was used with default parameters to log-transform the data and obtain the associated precision weights matrix. Limma with default parameters was applied to the resulting EList object to perform the differential expression analysis.

For all methods, Benjamini & Hochberg correction was used to adjust *p*-values for multiple testing (default in DESeq2 and Limma). Genes were called significantly differentially expressed when the program reported an adjusted *p*-value lower than 0.05.

R (v3.1.2) [70] was used to generate the correlation plots. The Venn diagrams were generated using jvenn [71]. IGV (v2.3) [72, 73] was used to visualize read coverage of the *Cp110* transcript and Mafft (v7.017) [74] (as integrated in Geneious v6.0.6 (Biomatters, Auckland, New Zealand)) was used to align the annotated *Cp110* transcripts of *D. melanogaster* and *D. mauritiana*.

### Real-time qPCR

RNA from eye-antennal imaginal discs from female LIII larvae was extracted using ZR Tissue & Insect RNA MicroPrep™ (Zymo Research, Irvine, CA, USA). RNA concentration was measured using Qubit (Invitrogen, Thermo Scientific, Waltham, Massachusetts, USA). Samples were diluted to contain exactly the same amount of starting RNA. RNA was converted to cDNA using MAXIMA® First Strand cDNA synthesis for RTqPCR (Thermo Scientific, Waltham, Massachusetts, USA). For the “no RT” control parallel reactions were carried out without enzyme. For the efficiency test, a series of five 1:4 dilutions were made. Real-Time qPCR was performed with HOT FIREpol ® EvaGreen® qPCR Mix Plus (ROX) (Solis BioDyne, Tartu, Estland) in a CFX96™ Real-Time PCR System (Bio-Rad Laboratories, Hercules, CA, USA). Primers were designed to exclude polymorphisms between *D. melanogaster* (FlyBase) and *D. mauritiana* TAM16 and to amplify a sequence that span introns to avoid genomic contamination (except for *Cp110*, *alrm* and *actin 79B*) and did not show isoform variation. Primer sequences are given in Additional file 10: Table S4. A melting curve was performed at the end of each reaction. Only genes that produced a single peak are shown. Expression differences were calculated by *log2*(2^-ΔΔCt^), using *actin 79B* as reference gene. Differences in expression were assessed using t-test/t-Welch-test with FDR = 0.05.

### Ethics statement

The research performed in this study on the fruit flies *Drosophila melanogaster*, *Drosophila mauritiana* and *Drosophila simulans* did not require approval by an ethics committee.

### Availability of data and materials

Raw fastq files of all samples have been deposited in NCBI’s Gene Expression Omnibus and are accessible through GEO Series accession number GSE76252 (http://www.ncbi.nlm.nih.gov/geo/query/acc.cgi?acc=GSE76252). A list of gene names (FBgn nomenclature) and the respective transcript lengths for all annotations used in this study (published annotations, direct re-annotation and the reciprocal re-annotation) of all three species are available as part of the processed files uploaded to the Gene Expression Omnibus (GEO) database (Accession number: GSE76252). Additional gff and fasta files of the final datasets and of intermediate steps of the reciprocal re-annotation pipeline are available from GSE76252 as well. All other additional files and figures are part of the “Additional files” of this publication.

## Additional files

Additional file 1: Table S1. Genes missing from the published annotations of *D. simulans* [53] and *D. mauritiana* [54] and from both species. (XLSX 123 kb) Additional file 2: Table S2. Raw values for the length differences of gene models between species. This table is the basis for Fig. 2. (DOCX 15 kb) Additional file 3: Figure S1.
*Correlation plots for DESeq2 using direct counts*. Relation between length differences and the log2-fold change. Comparisons between *D. mauritiana* and *D. melanogaster* are shown on the left side, comparisons between *D. mauritiana* and *D. simulans* are shown on the right side. On the first row, the published annotations are used as mapping references; on the second row, the directly re-annotated references are used as mapping references and on the third row, the reciprocally re-annotated references are used. Dots represent genes with length difference > 49 bp in these annotations. Genes significantly differentially expressed in the presented analysis (*padj* < 0.05) are shown in red. A negative log2-fold change indicates higher expression in *D. mauritiana.* A positive length difference indicates that the ortholog of *D. mauritiana* is longer. The *p*-value and rho of the Spearman’s rank correlation are indicated on the upper right side of the plots. (PDF 3164 kb) Additional file 4: Figure S2.
*Correlation plots for DESeq2 including length correction*. Relation between length differences and the log2-fold change. Comparisons between *D. mauritiana* and *D. melanogaster* are shown on the left side, comparisons between *D. mauritiana* and *D. simulans* are shown on the right side. On the first row, the published annotations are used as mapping references; on the second row, the directly re-annotated references are used as mapping references and on the third row, the reciprocally re-annotated references are used. Dots represent genes with length difference > 49 bp in these annotations. Genes significantly differentially expressed in the presented analysis (*padj* < 0.05) are shown in red. A negative log2-fold change indicates higher expression in *D. mauritiana.* A positive length difference indicates that the ortholog of *D. mauritiana* is longer. The *p*-value and rho of the Spearman’s rank correlation are indicated on the upper right side of the plots. (PDF 3389 kb) Additional file 5: Figure S3.
*Correlation plots for RPKM-limma*. Relation between length differences and the log2-fold change. Comparisons between *D. mauritiana* and *D. melanogaster* are shown on the left side, comparisons between *D. mauritiana* and *D. simulans* are shown on the right side. On the first row, the published annotations are used as mapping references; on the second row, the directly re-annotated references are used as mapping references and on the third row, the reciprocally re-annotated references are used. Dots represent genes with length difference > 49 bp in these annotations. Genes significantly differentially expressed in the presented analysis (*padj* < 0.05) are shown in red. A negative log2-fold change indicates higher expression in *D. mauritiana.* A positive length difference indicates that the ortholog of *D. mauritiana* is longer. The *p*-value and rho of the Spearman’s rank correlation are indicated on the upper right side of the plots. (PDF 3811 kb) Additional file 6: Figure S4.
*Correlation plots for RPKM-voom-limma*. Relation between length differences and the log2-fold change. Comparisons between *D. mauritiana* and *D. melanogaster* are shown on the left side, comparisons between *D. mauritiana* and *D. simulans* are shown on the right side. On the first row, the published annotations are used as mapping references; on the second row, the directly re-annotated references are used as mapping references and on the third row, the reciprocally re-annotated references are used. Dots represent genes with length difference > 49 bp in these annotations. Genes significantly differentially expressed in the presented analysis (*padj* < 0.05) are shown in red. A negative log2-fold change indicates higher expression in *D. mauritiana.* A positive length difference indicates that the ortholog of *D. mauritiana* is longer. The *p*-value and rho of the Spearman’s rank correlation are indicated on the upper right side of the plots. (PDF 3777 kb) Additional file 7: Figure S5.
*Cp110 coverage*. (A) Alignment of the published annotated transcripts of the gene *Cp110* in *D. mauritiana* (upper, shorter black bar), of the reciprocally re-annotated transcript in *D. mauritiana* (middle, long black bar) and of the transcript in *D. melanogaster* (lower, longer black bar; the published and the reciprocally re-annotated sequences are the same). Shades of grey indicate mismatches, the top ruler indicates the length of the alignment in bp, the green bar shows the base similarity. (B) *D. mauritiana* RNA-seq reads mapped to the body of the published *D. mauritiana Cp110* transcript. (C) *D. mauritiana* RNA-seq reads mapped to the body of the reciprocally re-annotated *D. mauritiana* Cp110 transcript. (D) *D. melanogaster* RNA-seq reads mapped to the body of the *D. melanogaster Cp110* transcript. Very few reads map to the 5′ region, more reads map from the central portion of the gene, and many more to the 3′ end. (PDF 409 kb) Additional file 8: Figure S6.
*Length difference of D. melanogaster gene models after reciprocal re-annotation*. The annotation of the *D. melanogaster* genome is considered to be the most complete and comprehensive one. After the reciprocal re-annotation of the *D. melanogaster, D. simulans* and *D. mauritiana* genomes the *D. melanogaster* gene models could be artificially truncated. This plot depicts the number of gene models that have X% of the original length after the reciprocal re-annotation. (PDF 271 kb) Additional file 9: Table S3. Mapping percentage of *D. melanogaster* replicate A to different references. (DOCX 12 kb) Additional file 10: Table S4. Sequences of the primers used for the qPCR experiment. (XLSX 11 kb)

## Abbreviations

BLAST: Basic Local Alignment Search Tool
Bp: base pairs
CDS: coding sequence
FDR: false discovery rate
qPCR: quantitative PCR
RPKM: reads per kilobase per million mapped reads
UTR: untranslated region
