# Supplementary material for: A robust (re-)annotation approach to generate unbiased mapping references for RNA-seq-based analyses of differential expression across closely related species
Source: BMC Genomics. 2016 May 24;17:392. doi: 10.1186/s12864-016-2646-x (PMC4877740; doi:10.1186/s12864-016-2646-x)
Supplement: Additional file 2: Table S2. — Raw values for the length differences of gene models between species. This table is the basis for Fig. 2. (DOCX 15 kb) [file 12864_2016_2646_MOESM2_ESM.docx]

| **Length difference** | ***D. melanogaster / D. mauritiana*** | ***D.melanogaster / D. simulans*** | ***D. mauritiana / D. simulans*** |
| --- | --- | --- | --- |
| **Published annotation** | |  |  |
| **0 bp** | 6,118 | 6,228 | 6,976 |
| **1 – 9 bp** | 2,043 | 1,773 | 1,527 |
| **10 – 49 bp** | 1,127 | 1,081 | 734 |
| **50 – 99 bp** | 239 | 495 | 378 |
| **100 – 499 bp** | 341 | 376 | 265 |
| **500 – 999 bp** | 71 | 27 | 62 |
| **1.000 - 9.999 bp** | 55 | 12 | 50 |
| **> 10.000 bp** | 0 | 2 | 2 |
| **Total** | **9,994** | **9,994** | **9,994** |
| **≤ 49 bp** | 9,288 | 9,082 | 9,237 |
|  |  |  |  |
| **Direct re-annotation** | |  |  |
| **0 bp** | 7,822 | 7,761 | 9,847 |
| **1 – 9 bp** | 3,414 | 3,456 | 2,046 |
| **10 – 49 bp** | 1,386 | 1,371 | 777 |
| **50 – 99 bp** | 143 | 142 | 123 |
| **100 – 499 bp** | 262 | 280 | 294 |
| **500 – 999 bp** | 147 | 151 | 125 |
| **1.000 - 9.999 bp** | 148 | 160 | 115 |
| **> 10.000** | 6 | 7 | 1 |
| **Total** | **13,328** | **13,328** | **13,328** |
| **≤ 49 bp** | 12,622 | 12,588 | 12,670 |
|  |  |  |  |
| **Reciprocal re-annotation** | |  |  |
| **0 bp** | 8,811 | 8,792 | 10,573 |
| **1 – 9 bp** | 3,238 | 3,368 | 2,190 |
| **10 – 49 bp** | 1,191 | 1,110 | 522 |
| **50 – 99 bp** | 28 | 17 | 12 |
| **100 – 499 bp** | 32 | 17 | 10 |
| **500 – 999 bp** | 6 | 3 | 3 |
| **1.000 - 9.999 bp** | 5 | 4 | 1 |
| **> 10.000** | 0 | 0 | 0 |
| **Total** | **13,311** | **13,311** | **13,311** |
| **≤ 49 bp** | 13,240 | 13,270 | 13,285 |
