# Supplementary material for: A robust (re-)annotation approach to generate unbiased mapping references for RNA-seq-based analyses of differential expression across closely related species
Source: BMC Genomics. 2016 May 24;17:392. doi: 10.1186/s12864-016-2646-x (PMC4877740; doi:10.1186/s12864-016-2646-x)
Supplement: Additional file 4: Figure S2. — Correlation plots for DESeq2 including length correction. Relation between length differences and the log2-fold change. Comparisons between D. mauritiana and D. melanogaster are shown on the left side, comparisons between D. mauritiana and D. simulans are shown on the right side. On the first row, the published annotations are used as mapping references; on the second row, the directly re-annotated references are used as mapping references and on the third row, the reciprocally re-annotated references are used. Dots represent genes with length difference > 49 bp in these annotations. Genes significantly differentially expressed in the presented analysis (padj < 0.05) are shown in red. A negative log2-fold change indicates higher expression in D. mauritiana. A positive length difference indicates that the ortholog of D. mauritiana is longer. The p-value and rho of the Spearman’s rank correlation are indicated on the upper right side of the plots. (PDF 3389 kb) [file 12864_2016_2646_MOESM4_ESM.pdf]

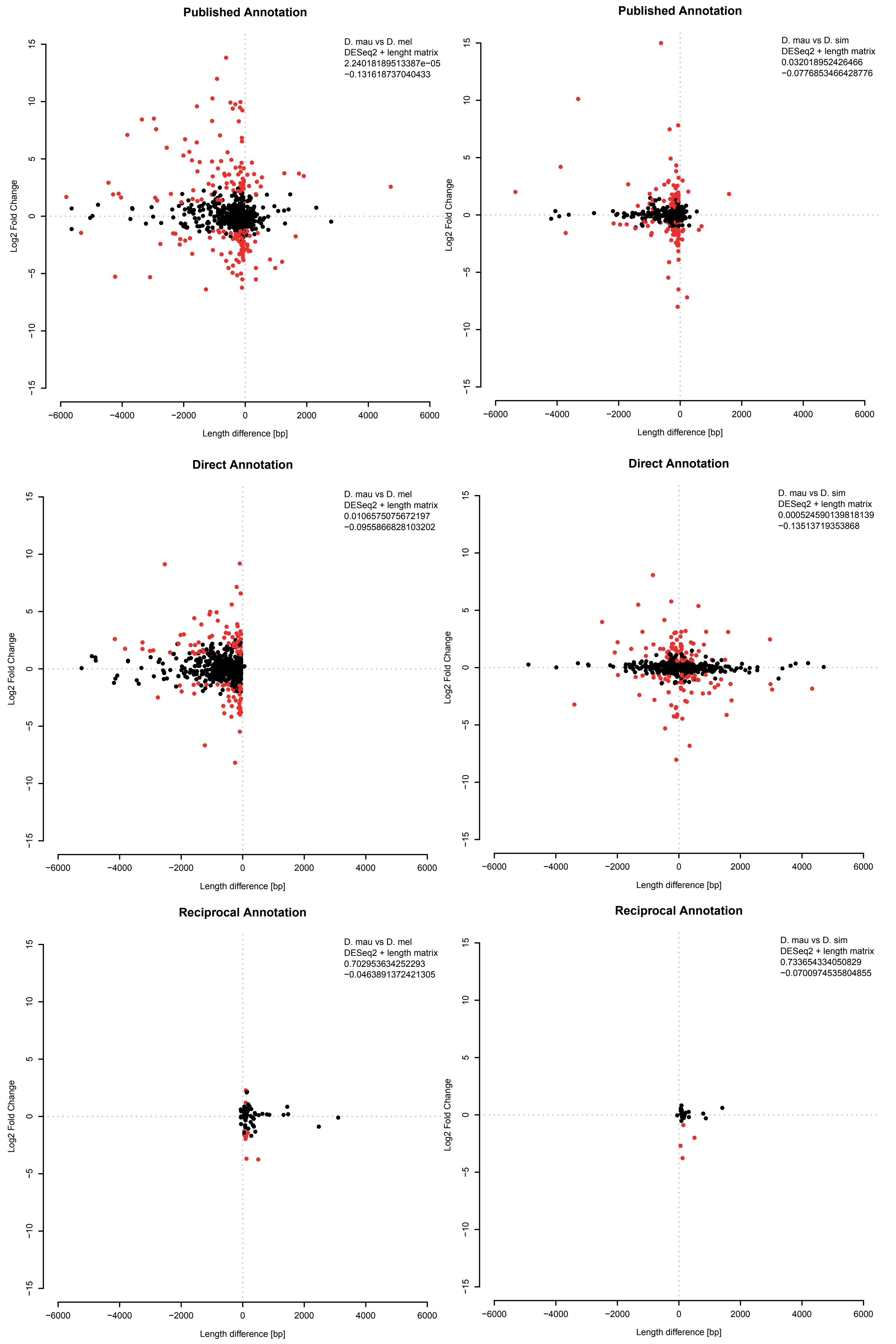

Published Annotation
D. mau vs D. mel
DESeq2 + lenght matrix
2.24018189513387e−05
−0.131618737040433
Log2 Fold Change
Length difference [bp]
Published Annotation
D. mau vs D. sim
DESeq2 + length matrix
0.032018952426466
−0.0776853466428776
Log2 Fold Change
Length difference [bp]
Direct Annotation
D. mau vs D. mel
DESeq2 + length matrix
0.0106575075672197
−0.0955866828103202
Log2 Fold Change
Length difference [bp]
Direct Annotation
D. mau vs D. sim
DESeq2 + length matrix
0.000524590139818139
−0.13513719353868
Log2 Fold Change
Length difference [bp]
Reciprocal Annotation
D. mau vs D. mel
DESeq2 + length matrix
0.702953634252293
−0.0463891372421305
Log2 Fold Change
Length difference [bp]
Reciprocal Annotation
D. mau vs D. sim
DESeq2 + length matrix
0.733654334050829
−0.0700974535804855
Log2 Fold Change
Length difference [bp]
