# Supplementary figures and images for: A robust (re-)annotation approach to generate unbiased mapping references for RNA-seq-based analyses of differential expression across closely related species
Source: BMC Genomics. 2016 May 24;17:392. doi: 10.1186/s12864-016-2646-x (PMC4877740; doi:10.1186/s12864-016-2646-x)

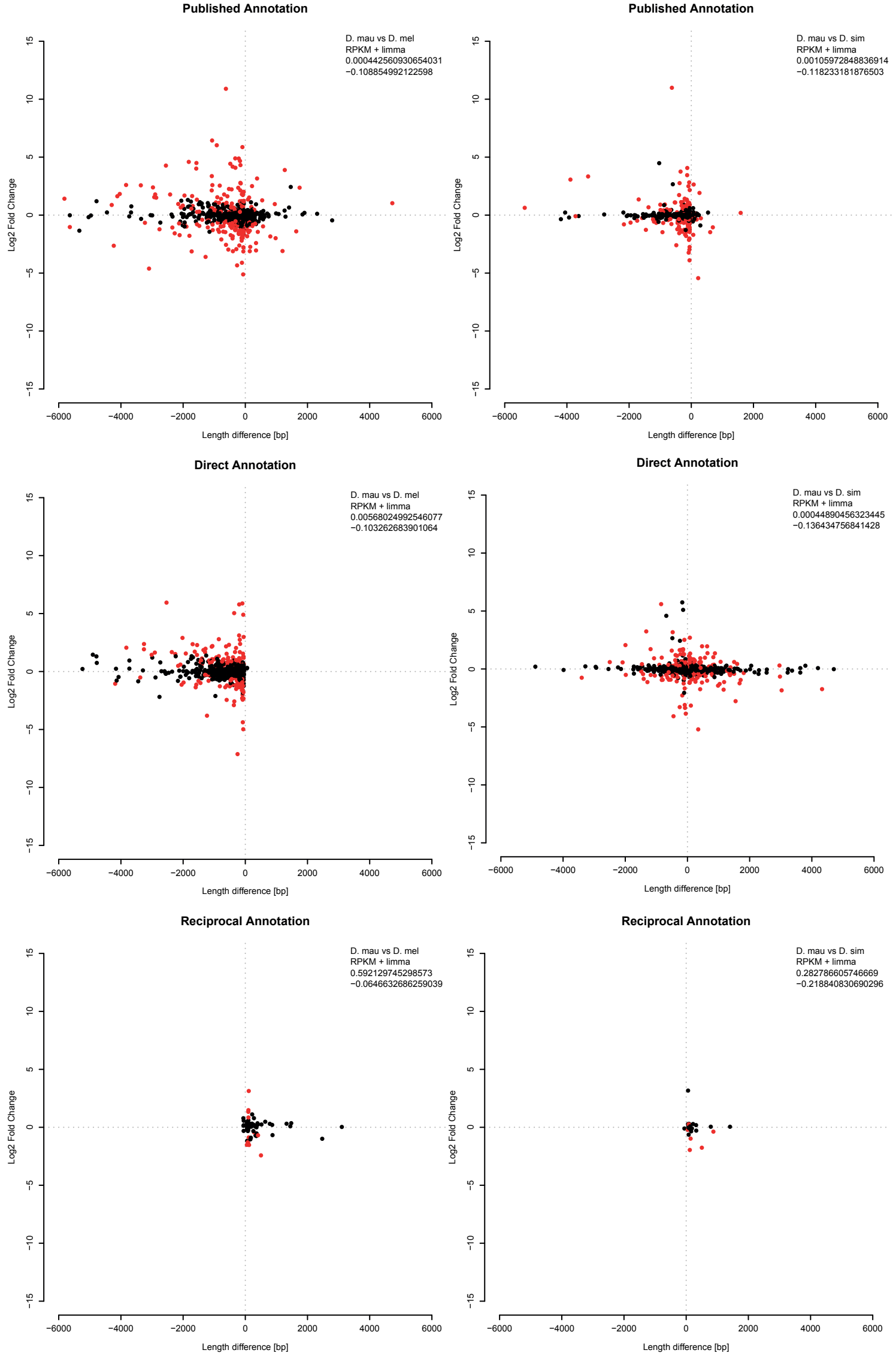

Supplement: Additional file 5: Figure S3. — Correlation plots for RPKM-limma. Relation between length differences and the log2-fold change. Comparisons between D. mauritiana and D. melanogaster are shown on the left side, comparisons between D. mauritiana and D. simulans are shown on the right side. On the first row, the published annotations are used as mapping references; on the second row, the directly re-annotated references are used as mapping references and on the third row, the reciprocally re-annotated references are used. Dots represent genes with length difference > 49 bp in these annotations. Genes significantly differentially expressed in the presented analysis (padj < 0.05) are shown in red. A negative log2-fold change indicates higher expression in D. mauritiana. A positive length difference indicates that the ortholog of D. mauritiana is longer. The p-value and rho of the Spearman’s rank correlation are indicated on the upper right side of the plots. (PDF 3811 kb) [file 12864_2016_2646_MOESM5_ESM.pdf]

Length compared to published annotation

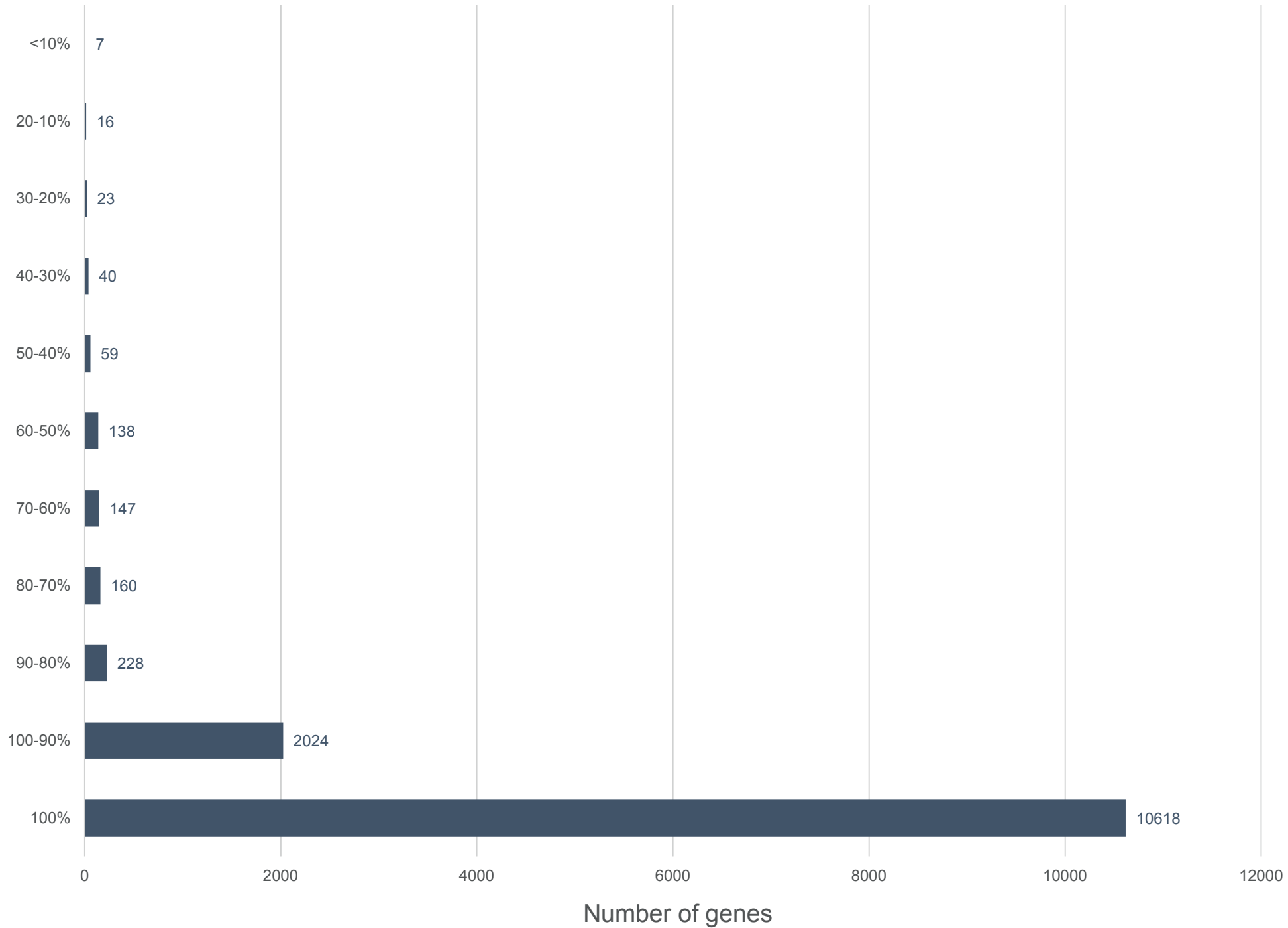

Supplement: Additional file 8: Figure S6. — Length difference of D. melanogaster gene models after reciprocal re-annotation. The annotation of the D. melanogaster genome is considered to be the most complete and comprehensive one. After the reciprocal re-annotation of the D. melanogaster, D. simulans and D. mauritiana genomes the D. melanogaster gene models could be artificially truncated. This plot depicts the number of gene models that have X% of the original length after the reciprocal re-annotation. (PDF 271 kb) [file 12864_2016_2646_MOESM8_ESM.pdf]
