# Supplementary material for: A robust (re-)annotation approach to generate unbiased mapping references for RNA-seq-based analyses of differential expression across closely related species
Source: BMC Genomics. 2016 May 24;17:392. doi: 10.1186/s12864-016-2646-x (PMC4877740; doi:10.1186/s12864-016-2646-x)
Supplement: Additional file 6: Figure S4. — Correlation plots for RPKM-voom-limma. Relation between length differences and the log2-fold change. Comparisons between D. mauritiana and D. melanogaster are shown on the left side, comparisons between D. mauritiana and D. simulans are shown on the right side. On the first row, the published annotations are used as mapping references; on the second row, the directly re-annotated references are used as mapping references and on the third row, the reciprocally re-annotated references are used. Dots represent genes with length difference > 49 bp in these annotations. Genes significantly differentially expressed in the presented analysis (padj < 0.05) are shown in red. A negative log2-fold change indicates higher expression in D. mauritiana. A positive length difference indicates that the ortholog of D. mauritiana is longer. The p-value and rho of the Spearman’s rank correlation are indicated on the upper right side of the plots. (PDF 3777 kb) [file 12864_2016_2646_MOESM6_ESM.pdf]

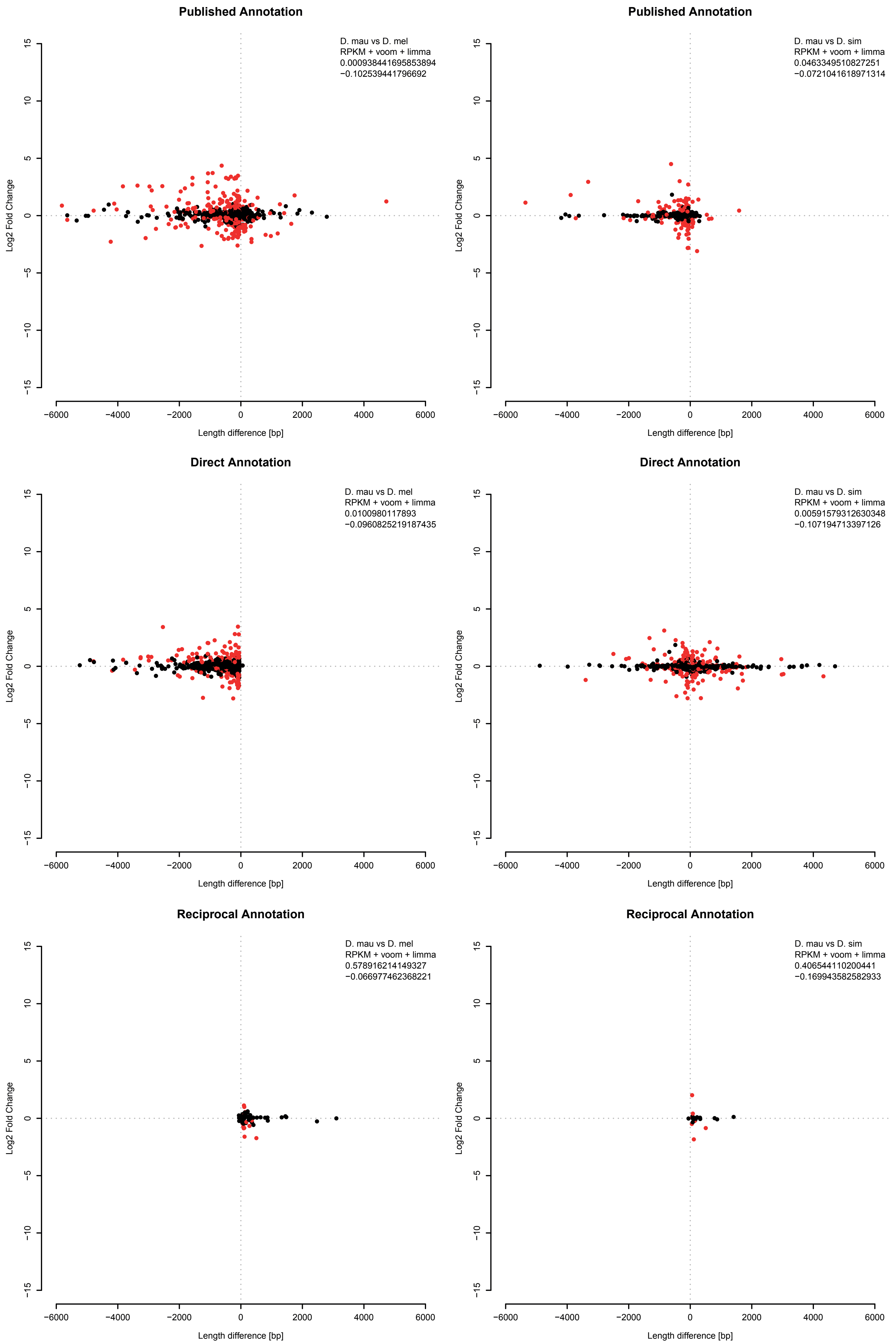
Published Annotation
D. mau vs D. mel
RPKM + voom + limma
0.000938441695853894
−0.102539441796692
Published Annotation
D. mau vs D. sim
RPKM + voom + limma
0.0463349510827251
−0.0721041618971314
Direct Annotation
D. mau vs D. mel
RPKM + voom + limma
0.0100980117893
−0.0960825219187435
Direct Annotation
D. mau vs D. sim
RPKM + voom + limma
0.00591579312630348
−0.107194713397126
Reciprocal Annotation
D. mau vs D. mel
RPKM + voom + limma
0.578916214149327
−0.066977462368221
Reciprocal Annotation
D. mau vs D. sim
RPKM + voom + limma
0.406544110200441
−0.169943582582933
Log2 Fold Change
Length difference [bp]
