# Supplementary material for: A robust (re-)annotation approach to generate unbiased mapping references for RNA-seq-based analyses of differential expression across closely related species
Source: BMC Genomics. 2016 May 24;17:392. doi: 10.1186/s12864-016-2646-x (PMC4877740; doi:10.1186/s12864-016-2646-x)
Supplement: Additional file 7: Figure S5. — Cp110 coverage. (A) Alignment of the published annotated transcripts of the gene Cp110 in D. mauritiana (upper, shorter black bar), of the reciprocally re-annotated transcript in D. mauritiana (middle, long black bar) and of the transcript in D. melanogaster (lower, longer black bar; the published and the reciprocally re-annotated sequences are the same). Shades of grey indicate mismatches, the top ruler indicates the length of the alignment in bp, the green bar shows the base similarity. (B) D. mauritiana RNA-seq reads mapped to the body of the published D. mauritiana Cp110 transcript. (C) D. mauritiana RNA-seq reads mapped to the body of the reciprocally re-annotated D. mauritiana Cp110 transcript. (D) D. melanogaster RNA-seq reads mapped to the body of the D. melanogaster Cp110 transcript. Very few reads map to the 5′ region, more reads map from the central portion of the gene, and many more to the 3′ end. (PDF 409 kb) [file 12864_2016_2646_MOESM7_ESM.pdf]

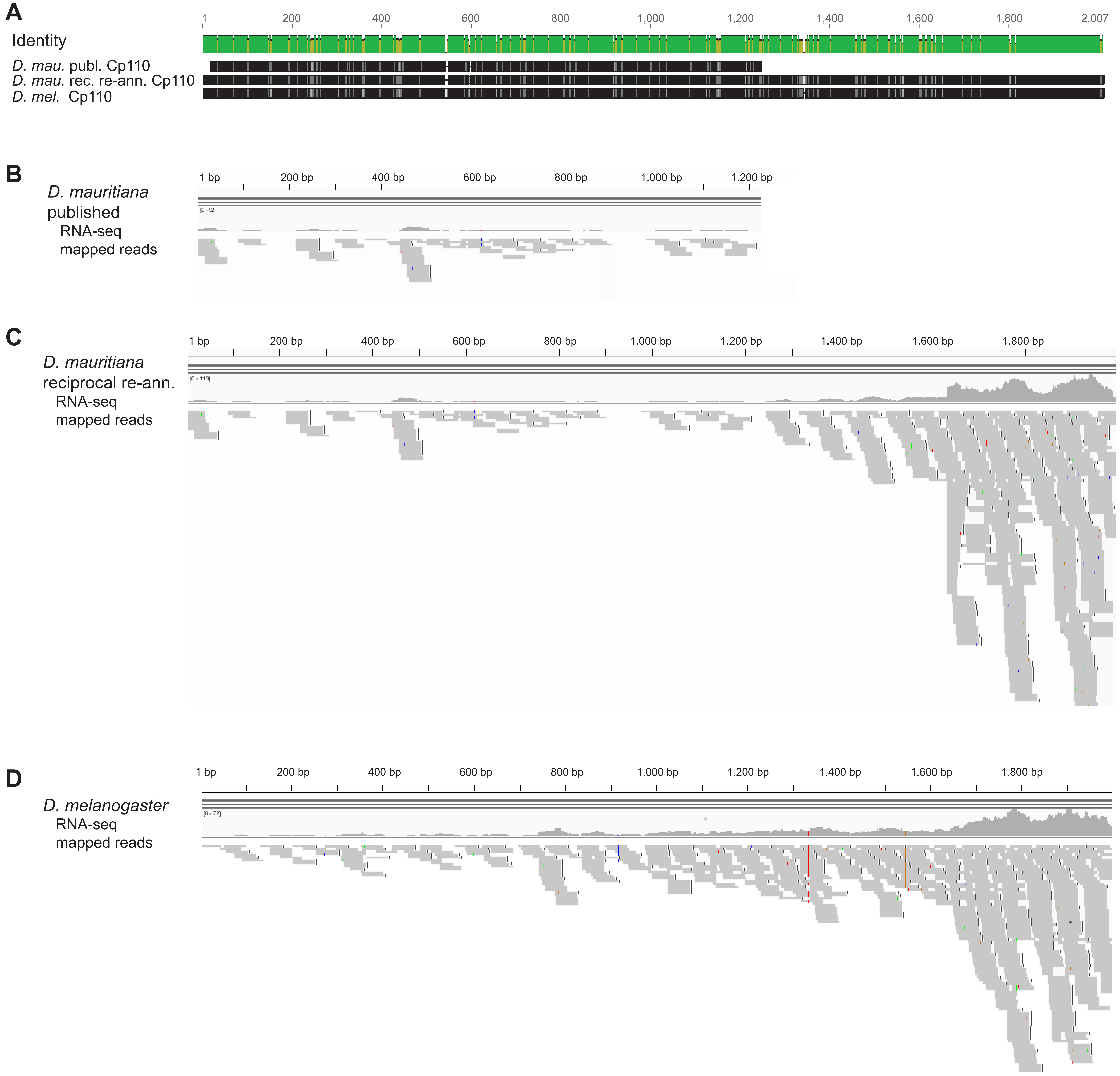
A
Identity
D. mau. publ. Cp110
D. mau. rec. re-ann. Cp110
D. mel. Cp110
1
200
400
600
800
1,000
1,200
1,400
1,600
1,800
2,007
B
D. mauritiana published
RNA-seq mapped reads
1 bp
200 bp
400 bp
600 bp
800 bp
1.000 bp
1.200 bp
C
D. mauritiana reciprocal re-ann.
RNA-seq mapped reads
1 bp
200 bp
400 bp
600 bp
800 bp
1.000 bp
1.200 bp
1.400 bp
1.600 bp
1.800 bp
D
D. melanogaster
RNA-seq mapped reads
1 bp
200 bp
400 bp
600 bp
800 bp
1.000 bp
1.200 bp
1.400 bp
1.600 bp
1.800 bp
