# Supplementary material for: A robust (re-)annotation approach to generate unbiased mapping references for RNA-seq-based analyses of differential expression across closely related species
Source: BMC Genomics. 2016 May 24;17:392. doi: 10.1186/s12864-016-2646-x (PMC4877740; doi:10.1186/s12864-016-2646-x)
Supplement: Additional file 9: Table S3. — Mapping percentage of D. melanogaster replicate A to different references. (DOCX 12 kb) [file 12864_2016_2646_MOESM9_ESM.docx]

| **Reference** | **aligned 0 times** | **aligned 1 time** | **aligned >1 times** | **overall alignment** |
| --- | --- | --- | --- | --- |
| **longest coding sequences** | 40.45% | 56.71% | 2.84% | 59.55% |
| **all coding sequences** | 40.03% | 18.84% | 41.13% | 59.97% |
| **longest full transcripts** | 14.24% | 80.14% | 5.61% | 85.76% |
| **all full transcripts** | 12.62% | 25.16% | 62.22% | 87.38% |
| **genome** | 4.79% | 86.97% | 8.24% | 95.21% |
